# Design and Validation of a Biomimetic Leg-Claw Mechanism Capable of Perching and Grasping for Multirotor Drones

**DOI:** 10.3390/biomimetics10010010

**Published:** 2024-12-27

**Authors:** Yan Zhao, Ruzhi Xiang, Hui Li, Chang Wang, Jianhua Zhang, Xuan Liu, Yufei Hao

**Affiliations:** 1 School of Mechanical Engineering, University of Science and Technology Beijing, Beijing 100083, China; yanzhao@ustb.edu.cn (Y.Z.); wangchang@ustb.edu.cn (C.W.); yufeihao@ustb.edu.cn (Y.H.); 2 School of Mechanical Engineering, Hebei University of Technology, Tianjin 300131, China; 202231205067@stu.hebut.edu.cn (R.X.); liuxuan@hebut.edu.cn (X.L.)

**Keywords:** aerial manipulator, biomimetic leg-claw mechanism, passive-active grasping, perching with tendon, gripping force analysis

## Abstract

Multirotor drones are widely used in fields such as environmental monitoring, agricultural inspection, and package delivery, but they still face numerous challenges in durability and aerial operation capabilities. To address these issues, this paper presents a biomimetic leg-claw mechanism (LCM) inspired by the biomechanics of birds. The claw of the LCM adopts a bistable gripper design that can rapidly close through external impact or actively close via the coordination of internal mechanisms. Additionally, its foldable, parallelogram-shaped legs bend under external forces, stretching the main tendon. A ratchet and pawl mechanism at the knee joint locks the leg in the bent position, thereby enhancing the gripping force of the claw. This paper calculates and experimentally verifies the degrees of freedom in different states, the forces required to open and close the gripper, the application scenarios of active and passive grasping, and the maximum load capacity of the mechanism. Furthermore, perching experiments demonstrate that the LCM enables the drone to perch stably on objects of varying diameters.

## 1. Introduction

The widespread use of drones in agriculture, logistics, construction, and infrastructure monitoring brings innovation and efficiency. However, drones often face challenges with limited endurance and maneuverability. Equipping drones with bird-like legs and claws would significantly enhance their flexibility and adaptability. Such drones could perch for extended monitoring, improving endurance, and use the leg-claw mechanism (LCM) for precise, swift deliveries, thereby enhancing their interaction with the environment.

Researchers have explored various perching methods for different scenarios. For flat, smooth surfaces, suction cup adhesion [1,2,3] and adhesive-based [4,5] methods are common. Suction cups provide strong adhesion but are prone to air leakage on rough surfaces. Adhesive methods also offer strong adhesion but lose stickiness over time and are affected by moisture. For rough and soft surfaces, biomimetic spiking methods [6,7,8] are suggested. However, its load capacity depends on the number of spines, their arrangement, and the load distribution. If the load is too heavy or the surface is uneven, the gripping force may be insufficient. External module mechanisms [9,10] are widely used but do not completely close the drone’s rotor, increasing energy consumption. Overall, current perching methods face challenges with surface condition limitations, load-bearing capacity, precision, and flexibility.

Researchers have explored various gripping mechanisms for aerial grasping. Mounting a robotic arm underneath the drone is the simplest method [11,12], using small servo systems to drive grippers that provide sufficient clamping force but add weight. Passive-triggered grippers achieve rapid closure due to a quick-release mechanism [13]. Guo et al. [14] explored the design of a flexible, self-adaptive gripper inspired by vine plants. Using a soft gripper for drones was another design approach [15]. However, they lack sufficient flexibility in certain tasks. These mechanisms often have singular functionalities, lacking universality and adaptability for simultaneous grasping and perching tasks.

Research into avian behavior has led to the development of mechanical clamp-type perching mechanisms based on avian biomimicry. These mechanisms enable drones to perch like birds, enhancing their flexibility and agility. Nagendran et al. [16] proposed a biologically inspired leg landing system using mechanical linkages and spring-damping materials to mimic bird legs, reducing impact and preventing slipping. Doyle et al. [17] designed a biomimetic mechanism with variable stiffness fingers and tendon-stretching legs for stable perching on irregularly shaped targets. Nadan et al. [18,19] developed landing gear with under-actuated multi-segment legs that use the drone’s weight to surround the target. Askari et al. [20] designed a bird claw with Hoberman link legs and Fin Ray claws for passive perching. While these studies demonstrate effective perching, they do not explicitly address grasping capabilities.

Researchers have developed systems combining biomimetic mechanical clamp-type perching and grasping mechanisms. Xie et al. [21] designed a system with cable-driven leg and finger mechanisms for passive grasping and perching. Zhang et al. [22] proposed a bistable gripper for drones that enables both perching and grasping. They further developed this concept [23], introducing a gripper with two perching methods—encircling and clipping—suitable for different objects, and demonstrated the clipping method’s effectiveness for grasping. Hsiao et al. [24] developed a passive gripper that closes on impact and opens upon weight removal. Liu et al. [25] designed a multi-finger system for landing on uneven terrain and grasping objects. Despite their capabilities, these mechanisms are limited in speed. McLaren et al. [26] introduced a quick-release robotic hand with high-speed grasping using distance sensors. Roderick et al. [27] proposed SNAG, a bionic bird leg mechanism that adapts to irregular surfaces. However, compared to our mechanism, it employs a passive grasping form, making it difficult to actively contract or release the gripping device through its actions, which limits its application range in non-cooperative target grasping. Firouzeh et al. [28] developed a bionic passive dynamic gripper driven by impact energy, featuring a soft tendon for energy absorption and rapid closure. However, its inability to apply additional gripping force once closed can lead to perching instability.

As mentioned above, while significant advancements have been made in rotor drone operating arms and takeoff/landing devices, developing a drone mechanical leg-claw system with the capability of flexible grasping and self-locking perching remains a challenge. Therefore, the purpose of developing the LCM is to enhance the functionality and performance of drones by integrating biomimetic principles inspired by bird perching and grasping mechanisms. By mimicking the precise control of bird legs and claws, the LCM design enables both active and passive grasping capabilities, ensuring improved stability and adaptability in various operational conditions. Additionally, the mechanism aims to enhance drone endurance by reducing energy consumption during perching, enabling long-duration monitoring and mission execution. Ultimately, the development of the LCM seeks to expand the potential applications of drones in complex environments, such as rescue operations and precision agriculture, by offering grasping and perching abilities that traditional systems cannot achieve.

In this paper, our main contributions are as follows:

1. We propose an innovative biomimetic LCM to address the aforementioned challenges. This LCM enables the drone to achieve perching functionality, with both active and passive grasping capabilities, and it can maintain these states without additional energy input.

2. Through simulations and experimental validation, we demonstrate that the biomimetic LCM exhibits superior performance in load capacity, grasping success rate, and adaptability when grasping different objects, highlighting its effectiveness in diverse operational conditions. Additionally, the LCM’s perching capability further enhances its performance by providing stable support on various surfaces.

The rest of this paper is organized as follows: Section 2 describes and analyzes the structural composition, working principles, and degrees of freedom (DoFs) of the LCM. Section 3 establishes the static model of the LCM gripper during the opening phase, determining the gripping space and conducting a static analysis to ascertain the maximum load the mechanism can handle. Section 4 measures the forces during the gripper’s opening process and the force required to trigger its closure. Additionally, experiments are conducted to compare the success rates of active and passive grasping for different objects and to verify the accuracy of the static model for object gripping. Furthermore, the LCM’s ability to grasp objects and assist drones in perching is demonstrated.

## 2. Design and Analysis of Mechanism

### 2.1. Biomimetic Structural Design

**Figure 1:**
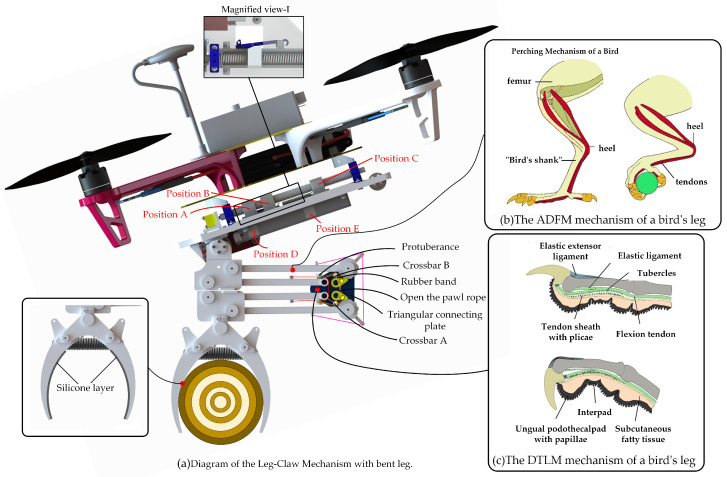
(**a**)The overall bionic leg-claw mechanism of the drone. (**b**) The ADFM mechanism of a bird’s leg. (**c**) The DTLM mechanism of a bird’s leg [31,32].

Based on the anatomical mechanisms of bird perching studied by researchers, the key features of bird perching and grasping lie in efficient mechanical force transmission, tendon locking mechanisms, and precise neuromuscular control. These mechanisms allow birds to firmly grasp objects and maintain their grip during perching without expending excessive energy [29], as shown in Figure 1b.

The legs and claws of birds consist of the femur, tibia, fibula, and tarsometatarsus, which together form a powerful support and grasping system. The primary active joints in bird claws are the toe joints, which are typically curved, allowing them to grasp and wrap around objects. The motion of bird claws is primarily driven by flexor and extensor muscles. Bird grasping mechanisms are divided into active and passive grasping mechanisms.

During active grasping, the flexor muscles contract, causing the toe joints to bend and form a gripping action. The extensor muscles are responsible for releasing the grip. When the extensor muscles relax, the claws open, releasing the object. Active grasping is typically used to catch and hold objects, such as when birds capture prey, allowing for precise control of the grip to avoid excessive harm to the prey.

The passive grasping mechanism, on the other hand, is a natural mechanism used by birds during perching, which does not rely on continuous muscle contraction but instead uses tendon locking to maintain the grip. The stretching and locking of tendons are mainly driven by the “Automatic Perching Mechanism” (APM) [30]. This mechanism not only ensures that birds can stand securely on perches but also reduces energy consumption. Even when birds are resting or sleeping, their claws can maintain a stable grip on the perch to prevent falling. The APM consists of the Automatic Digital Flexor Mechanism (ADFM) and the Digital Tendon Locking Mechanism (DTLM). When birds stand or perch, the bending of the legs triggers the ADFM, which converts external pressure into gripping force in the claws. Subsequently, the DTLM mechanism engages, with small protrusions on the tendon interlocking with rigid ribs on the inner surface of the tendon sheath, locking the tendon and maintaining the bent position of the leg, thereby ensuring stable gripping force [31,32], as shown in Figure 1b,c.

Although birds primarily rely on tendon tension and locking mechanisms for passive grasping, friction plays a crucial supplementary role. The inner surfaces of bird claws are typically covered with soft tissues, which allow them to closely conform to the surface of the target object, increasing the contact area. A larger contact area helps distribute the pressure and significantly enhances the effectiveness of friction. Additionally, the flexibility and curvature of bird claws enable them to better adapt to the shape of the target object. Particularly when grasping irregular surfaces, this adaptability ensures higher contact friction and improved grasping success rates.

**Figure 2:**
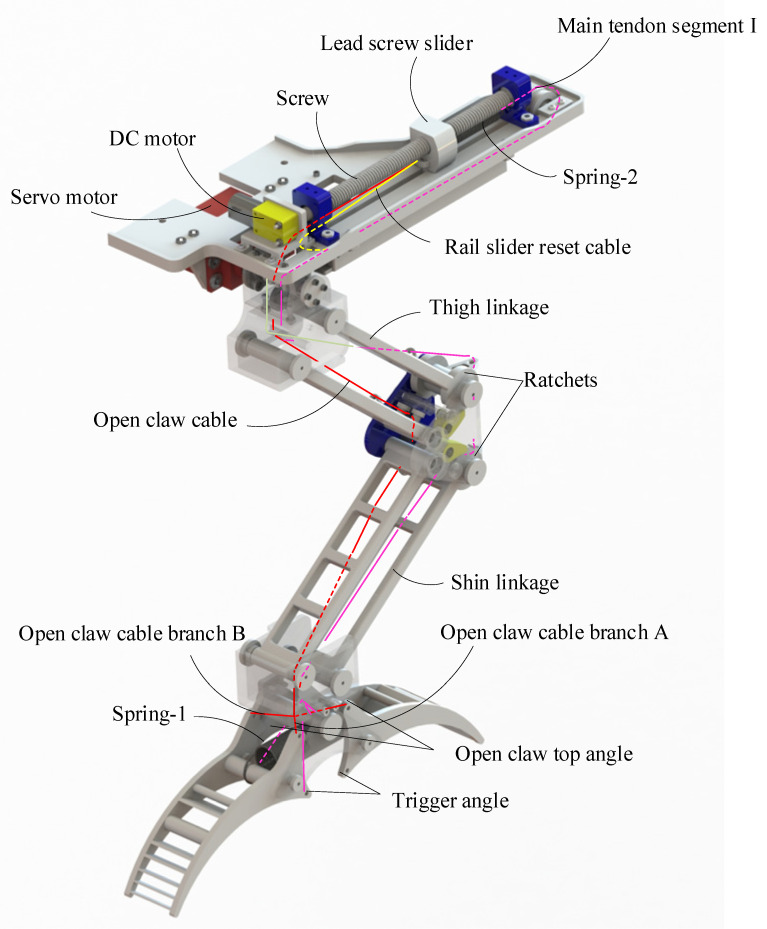
Front view diagram of the leg-claw mechanism.

In our research, the design of the quadrotor drone leg-claw mechanism is inspired by the bionic study of bird leg structure and movement, as shown in Figure 2. The leg structure consists of two parallel four-bar linkages, which fold up and down, enhancing the leg’s flexibility, evenly distributing the load, and providing stronger support and stability. The claw adopts a simplified bistable gripper design. The core drive system of the entire LCM is made up of springs and cables, allowing for quick switching between grasping and releasing. The LCM is capable of passive grasping and perching actions triggered by external forces, as well as active grasping through the coordination of its internal components. This design mimics the core principles of bird perching and grasping mechanisms.

Additionally, our design incorporates a dual-pawl ratchet mechanism at the knee joint. When the leg bends and stretches the tendon, the dual-pawl ratchet mechanism locks, maintaining the bent position of the leg and thereby preserving the tension in the tendon, which in turn maintains the grasping force of the LCM. This mechanism is similar to the Automatic Perching Mechanism (APM) in birds. In our design, a layer of silicone was added to the claws, mimicking the functional properties of bird claw soft tissues.

Other design details include a servo motor at the hip joint, which adjusts the leg angle and helps balance the drone during perching. A DC motor above the hip drives the forward and backward movement of the screw slider, allowing the gripper to open and close, while storing energy in spring-2 during motion. Below the hip, a rail and rail-slide mechanism with a slide rail latch secures the rail slide, storing the energy required for tendon retraction in spring-2.

### 2.2. Analysis of Mechanism Motion Process

In Figure 1 and Figure 2, the primary tendon is illustrated by the pink line, which extends throughout the hip and leg and is divided into two sections. The first section connects from spring-2 to the rear of the rail slider (with the front oriented towards the hip joint). The second section runs from the front of the rail slider along the outer side of the leg link to the claw, attaching to the four trigger angles of the claw. The red line represents the open-claw rope, which links the front of the screw slider to the claw along the inner side of the leg link, connecting to the four top angles for opening the claw. The yellow line indicates the rail slider reset rope, which connects the front of the rail slider to the front of the screw slider. Below the plate, the green line signifies the slide rail latch release rope, which functions to open the slide rail latch and release the track slider during grasping or perching. At the latch, a rubber band is connected to the tail of the slide rail latch, applying elastic force that exerts a clockwise torque to maintain the latch in a closed position. At the knee joint, the orange line represents the rope for opening the pawl, with one end connected to the pawl and the other end attached to the open-claw rope. The brown section symbolizes the rubber band, which is looped above the pawl and around the protrusion of the shin link. This rubber band generates torque to keep the pawl engaged with the ratchet, thereby locking the leg during impact and bending.

**Figure 3:**
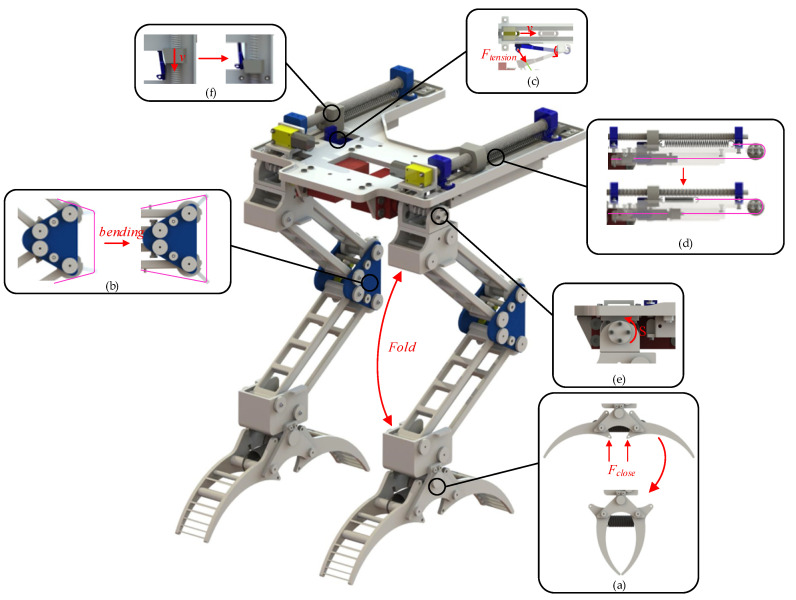
Detailed movement diagram of the LCM. (**a**) Trigger angle is activated by impact to initiate grabbing. (**b**) Leg bending to stretch the tendons. (**c**) The latch unlocks under the action of tension. (**d**) The slider moves along the rail, stretching the main tendon under the tension of spring-2. (**e**) The servo motor rotates to adjust the leg. (**f**) Lead screw slider movement, unlock the latch.

Above the hip, the DC motor rotates the screw, resulting in the reciprocation of the screw slider. Clockwise rotation of the motor moves the screw slider backward, away from the leg. As illustrated in Figure 2, the red rope tightens when the screw slider is positioned at C, opening the claw into its first stable state. Concurrently, under the tension of the yellow rope, the rail slider shifts from position E to position D, where it is secured by the slide rail latch, as shown in Figure 1. At the knee joint, the pawls disengage from the ratchets on the thigh and shin, permitting the leg to extend from a bent state to an extended state. Subsequently, the DC motor reverses, causing the screw slider to advance to position B. Due to the bistable mechanism, the claw remains open. As the screw slider progresses forward, spring-2 stretches. At this stage, LCM completes the preparation for grasping and perching, as depicted in Figure 3.

**Figure 4:**
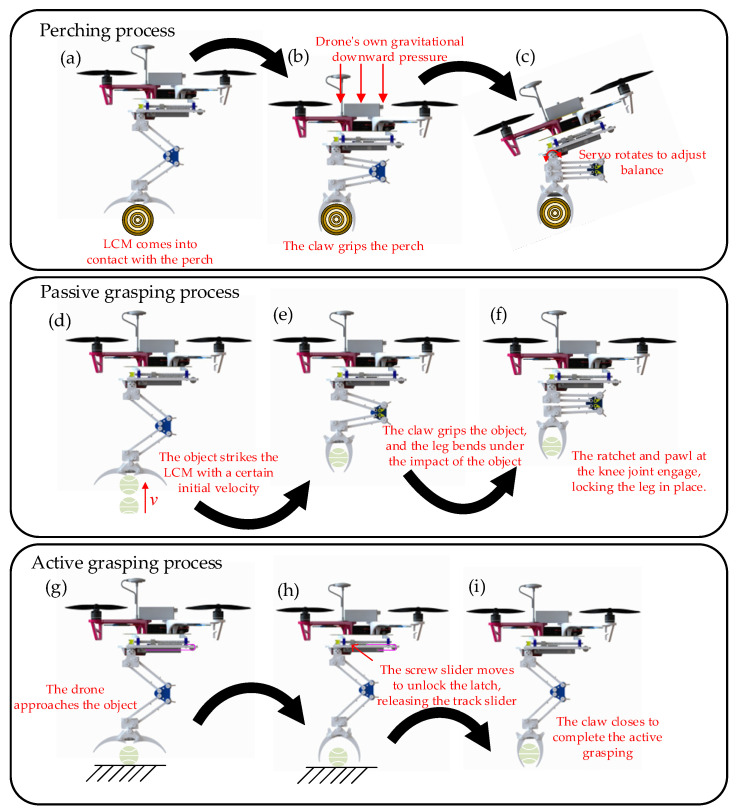
The transition process of the three motion modes of the LCM. (**a**) Perching preparation state. (**b**) During perching. (**c**) Completed perching state. (**d**) Passive grasping preparation state. (**e**) During passive grasping. (**f**) Passive grasping completed. (**g**) Active grasping preparation state. (**h**) During active grasping. (**i**) Active grasping completed.

During perching tasks, the drone descends toward the pole. Upon contact, the triggering corners of the claw disrupt its initial stable state, prompting rapid closure to grip the pole, as shown in Figure 3a and Figure 4a. As the drone continues to descend, gravitational forces cause the thigh and shin of LCM to fold, maintaining a bent position. This motion stretches the main tendon rope at the knee joint, as shown in Figure 3b. Concurrently, the slide rail latch release rope located beneath the hip also stretches, resulting in the opening of the slide rail latch, as shown in Figure 3c. With the slide rail latch released, the rail slider moves backward along the rail due to the force exerted by spring-2. This movement further tensions the main tendon, thereby increasing the gripping force of the claw, as shown in Figure 3d. Once perched, the servo motors at the hip joint adjust the balance of both the drone and the LCM, as shown in Figure 3e and Figure 4c.

The gripping mechanism of the drone’s LCM is categorized into passive gripping and active gripping. Passive gripping, similar to perching actions, entails the gripper rapidly closing upon contact with an object, thereby disrupting its initial stable state, as shown in Figure 4d. Concurrently, the leg of the LCM bends in response to the external force resulting from the object’s impact, while the pawls and ratchets of the knee joint engage to maintain the bent leg position, as shown in Figure 4f. Throughout this process, the main tendon and the rope connected to the slide rail latch are subjected to stretching. The tension in the rope triggers the opening of the slide rail latch, which enables the rail slider to further tighten the main tendon with the assistance of spring-2, thereby enhancing the gripping force.

The active gripping mechanism of the LCM functions independently of external impact forces by employing the spring force of spring-2, located above the hip, to close the gripper during grasping. Reversing the DC motor transitions the screw slider from position-B to position-A. The wedge-shaped design of the screw slider and slide rail latch enables the screw slider at position-A to elevate the latch, thereby unlocking it, as shown in Figure 3f. As a result, the rail slider rapidly moves toward position-E along the rail, driven by the action of spring-2, which tightens the main tendon and facilitates the swift closure of the claw, thereby achieving active gripping. The active grasping process of the LCM is shown in Figure 4g–i.

### 2.3. Analysis of Mechanical DoFs

The DoFs of the LCM dictate its flexibility and adaptability in executing various tasks. The LCM is composed of several components, including the frame, the hip joint connector, links, the knee joint connector, links, the claw connector, and the gripper. It features multiple revolute pairs (*R*1 to *R*11) and planar pairs (*RP*1 and *RP*2). The overall freedom of the mechanism is contingent upon the position of the hip screw slider, as illustrated in Figure 1.

**Figure 5:**
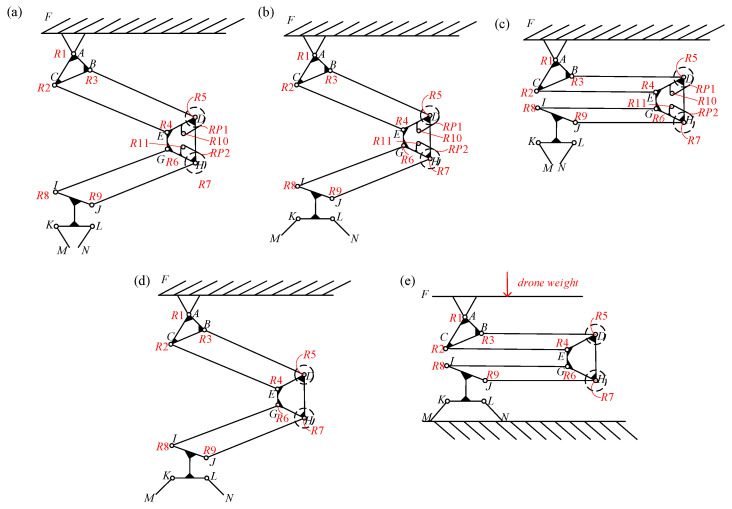
Schematic of the degree of freedom calculation for the mechanism. (**a**) LCM in an active gripping state with natural leg extension. (**b**) LCM in gripping preparation state with natural leg extension. (**c**) LCM in passive gripping state with bent leg. (**d**) LCM in the naturally extended state. (**e**) Status of LCM when used as landing gear.

At Position A, the screw slider disengages the slide rail latch, allowing the rail slider to move from Position D to Position E along the rail due to the tension in spring 2. This movement tightens the main tendon, which restricts leg bending and transitions the claw from an open to a closed position. The force exerted by spring 2 enhances the claw’s gripping strength while limiting its rotational freedom (Figure 5a). Consequently, the mechanism possesses one DoF.

At position B, with the leg naturally extended, the rail slider is located at position D, where the main tendon is relaxed, spring 2 is stretched, and the claw is open. The ratchet and pawl mechanism at the knee joint is engaged (Figure 5b). According to Equation (1), the leg has three DoFs, while the gripper has one DoF, yielding a total of four DoFs.

At position B, with the leg fully bent due to external impact, the pawls and ratchets of the knee joint secure the leg in a bent position. As the leg bends, the claw closes, the main tendon tightens, and the latch of the slide rail unlocks. Subsequently, Spring 2 moves the rail slider from position D to position E, further closing the claw. The only remaining DoF in the LCM is the rotation of the hip joint. The schematic of the mechanism is illustrated in Figure 5c, indicating a total of one degree of freedom.

At position C, with the leg naturally extended, the ratchets and pawls of the knee joint disengage. The revolute pairs *R12* and *R13*, along with the higher pairs *RP1* and *RP2*, become inactive. The open claw rope remains taut, maintaining the claw’s open position and constraining its degrees of freedom (DoFs). A simplified diagram of the mechanism is presented in Figure 5d. According to Equation (1), the total degrees of freedom for the entire mechanism is calculated to be three.

At position C, the claws of the LCM remain in an open state.

Leg bending requires external force for initiation and maintenance, making this configuration suitable as landing gear for drones. In this scenario, the mechanism has zero overall DoFs. The schematic diagram of the entire mechanism is shown in Figure 5e.

(1) $$F=3n-2p_{l}-p_{h}$$

where *F* represents the number of degrees of freedom of the organization, n represents the number of active components, $p_{l}$ represents the number of lower pairs, and $p_{h}$ represents the number of higher pairs.

## 3. Static Modeling of LCM

### 3.1. Force Required to Open the Dual Stable Gripper

In the LCM, the key to opening the claw is to ensure that the torque exerted by the tension in the opening rope on the rotation center *O* is greater than the torque exerted by spring-1 on the rotation center *O* during the gripper opening process. However, this process involves a series of complex issues, including the continuous change in the direction of the rope tension as the gripper opens. Therefore, it is necessary to establish a static force model of the gripper to determine the force required to open the claw.

**Figure 6:**
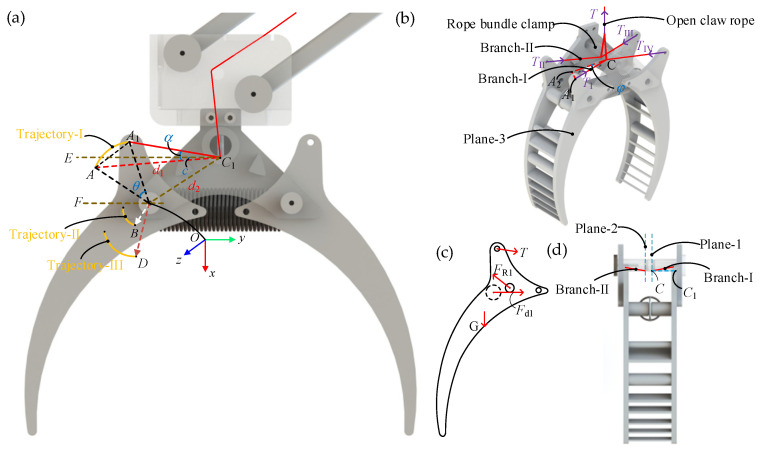
Illustration of the claw opening process. (**a**) Geometric relationship diagram during the opening process of the gripper. (**b**) Schematic diagram of the direction of rope tension. (**c**) Force analysis of the left gripper. (**d**) Schematic diagram of the location of the planes.

As the screw slider moves from position-A to position-C, the claw transitions from closed to open. During this change, the four branches of the opening claw rope are labeled as I, II, III, and IV. On one side of the gripper, it experiences tension from two branches of the rope, as shown in Figure 6b. We focus on analyzing the forces acting on one side of the gripper, as depicted in Figure 6c.

To determine the angle variation of the branches of the opening claw rope during the claw opening process, three planes were created for calculations, depicted in Figure 6d. Plane-1 passes through the centerline of the small hole on the rope bundle clamp and is parallel to the *xy*-Plane. Plane-3 represents the surface of the gripper, also parallel to the *xy*-Plane. Point *C* resides on Plane-1, while point *O* is on Plane-3, and *C*_1_ is the projection of *C* onto Plane-3. *d*_3_ represents the distance between Plane-1 and Plane-3. Let φ denote the angle between branch-I and Plane-1 during the gripper opening process. The positions, angles, and trajectories of key points during the gripper’s opening are illustrated in Figure 6a, projected onto Plane-3. Trajectory-I shows the path of vertex *A* during rotation with a radius of $r_{1}$. Trajectory-II represents the trajectory of the center of axis *B*, where spring-2 is mounted, with a radius of $r_{2}$. Trajectory-III tracks the gripper’s center of mass *D* during rotation, with a radius of $r_{3}$. *AC*_1_ projects branch-I onto Plane-3 when the gripper is closed, and *A*_1_*C*_1_ is its projection in the current gripper state. Let α represent the angle between *AC*_1_ and *A*_1_*C*_1_, and *C*_1_*E* be an auxiliary line parallel to the *y*-axis. The angle between *AC*_1_ and *C*_1_*E* is denoted as c, and *d*_1_ is the length between *AC*_1_. *OA* and *OA*_1_ are connected to represent the gripper’s rotation angle, denoted as θ. *OF* is an auxiliary line through *O* parallel to the *y*-axis, and the angle between *OA* and *OF* is denoted as *a*. *OC*_1_ is the line connecting *O* and *C*_1_ with a length of *d*_2_, and *e* is the angle between *OC*_1_ and the extension line of *OF*. The angle between *OB* and *OF* is denoted as *b*, and the angle between *OD* and *OF* is denoted as *i*. *d*_3_ represents the distance between Plane-1 and Plane-3.

During this portion of the static analysis, the following assumptions were consistently made:

Friction at the contact point between the rope and the LCM is negligible when the rope is stretched.

The length of the rope remains nearly constant when it is stretched.

The length of the spring changes according to Hooke’s Law.

During the opening process of the gripper, the forces exerted by branches I and II on the gripper, as well as the torque direction at point *O*, change with the rotation angle θ. Through calculation, they can be classified into the following two cases:

When $\theta <\frac{\pi }{2}-a$, the torque equation at point *O* after resolving the force of branch-I is

(2) $$M_{O}=-2T_{I}\cdot \cos\varphi \cdot r_{1}\cdot \sin\left(a+\theta -\alpha +c\right)+G\cdot r_{3}\cdot \cos\left(i-\theta \right)+F_{d1}\cdot r_{2}\cdot \sin\left(b-\theta \right)$$

where $F_{d1}$ represents the spring force of spring-1, $T_{I}$ represents the tension of branch-I and G represents the gravitational force of the gripper.

When $\theta >\frac{\pi }{2}-a$, the torque equation at point *O* after resolving the force of branch-I is

(3) $$M_{O}=-2T_{I}\cdot \cos\varphi \cdot r_{1}\cdot \sin\left(a+\theta +\alpha -c\right)+G\cdot r_{3}\cdot \cos\left(i-\theta \right)+F_{d1}\cdot r_{2}\cdot \sin\left(b-\theta \right).$$

In the triangle formed by points *A*, *A*_1_, and *C*_1_, applying the cosine rule, we obtain

(4) $$\alpha =\arccos\left(\frac{d_{1}^{2}+r_{1}^{2}+d_{2}^{2}+2r_{1}\cdot d_{2}\cdot \cos\left(a+\theta +e\right)-2r_{1}^{2}\left(1-\cos\theta \right)}{2\cdot d_{1}\cdot \sqrt{r_{1}^{2}+d_{2}^{2}+2r_{1}\cdot d_{2}\cdot \cos\left(a+\theta +e\right)}}\right).$$

The length of *A*_1_*C* is approximately equal to the length of rope branch-I. From Figure 4b), using the Pythagorean theorem, the length of *A*_1_*C* is derived as

(5) $$A_{1}C=\sqrt{d_{3}^{2}+r_{1}^{2}+d_{2}^{2}+2r_{1}\cdot d_{2}\cdot \cos\left(a+\theta +e\right)}.$$

The angle φ between rope branch-I and Plane-3 can be obtained from Equation (5) using the cosine formula,

(6) $$\varphi =\arccos\left(\frac{\sqrt{r_{1}^{2}+d_{2}^{2}+2r_{1}\cdot d_{2}\cdot \cos\left(a+\theta +e\right)}}{\sqrt{d_{3}^{2}+r_{1}^{2}+d_{2}^{2}+2r_{1}\cdot d_{2}\cdot \cos\left(a+\theta +e\right)}}\right).$$

By deriving α and φ and converting them into expressions in terms of θ, the relationship between the gripper’s rotation angle and the direction change of rope branch-I can be determined.

When $\theta <\frac{\pi }{2}-a$, the magnitude of $T_{I}$ is determined to be

(7) $$T_{I}>\frac{G\cdot r_{3}\cdot \cos\left(i-\theta \right)+F_{d1}\cdot r_{2}\cdot \sin\left(b-\theta \right)}{\cos\varphi \cdot r_{1}\cdot \sin\left(a+\theta -\alpha +c\right)}.$$

When $\theta >\frac{\pi }{2}-a$, the magnitude of $T_{I}$ is determined to be

(8) $$T_{I}>\frac{G\cdot r_{3}\cdot \cos\left(i-\theta \right)+F_{d1}\cdot r_{2}\cdot \sin\left(b-\theta \right)}{\cos\varphi \cdot r_{1}\cdot \sin\left(a+\theta +\alpha -c\right)}.$$

By the above equation, the magnitude of $T_{I}$ is determined for different values of θ.

### 3.2. Analysis of the Grasping Range of the LCM

**Figure 7:**
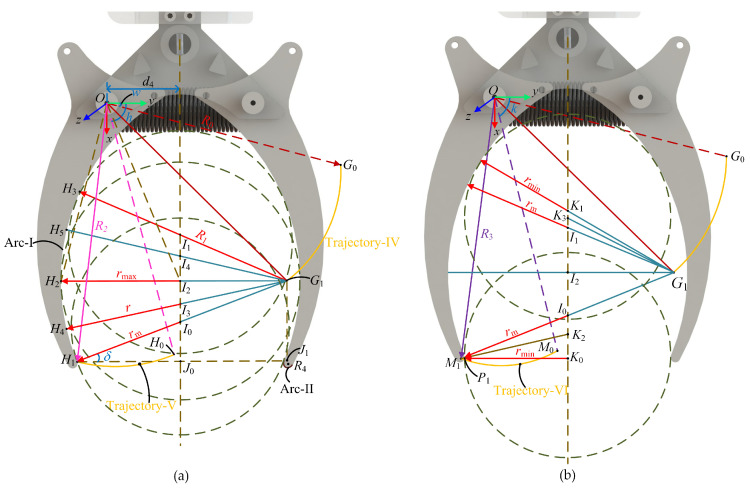
(**a**) Geometry diagram of the gripper and object when the contact point between the object and the gripper is located on Arc-I. (**b**) Geometry diagram of the gripper and object when the gripper grasps the object; the contact point between the object and the gripper is located on Arc-II.

A Cartesian coordinate system with the rotation center *O* as the origin is used to study the geometric characteristics of the gripper, as shown in Figure 7a. The inner wall of the gripper consists of Arc-I and Arc-II. Arc-I, centered at point *G*_0_ with radius $R_{1}$, follows Trajectory-IV around point *O*. Arc-II, centered at point *M*_1_ with radius $R_{4}$, and the rotating radius around point *O* is $R_{3}$. The intersection of the arcs is point *H*_1_, with Trajectory-V representing the motion of point *H*_1_ around *O*. The circles in the figure represent different object-gripping scenarios, with center positions labeled *I*_0_, *I*_1_, and *I*_2_. The maximum object radius, $r_{\max}$, occurs when the object’s center aligns with the center of Trajectory-IV. The circle centered at *I*_2_ represents this maximum radius when the gripper rotates by angle θ. The gripper’s inner wall always tangentially contacts the object’s contour during gripping. The minimum radius for Arc-I gripping, $r_{\min}$, occurs when the object is tangent to point *H*_1_. Objects between points *H*_1_ and *H*_2_ have a radius between $r_{\min}$ and $r_{\max}$. For Arc-II, objects with a radius less than $r_{\mathrm{mid}}$ are grasped. Figure 7b shows that when the tangent point is *P*_1_, the minimum graspable radius $r_{\min}$ is achieved when the object’s center aligns with the center of Arc-II.

Let the angle between *OG*_0_ and the *y*-axis be w, the angle between *OH*_0_ and the *y*-axis be h, the angle between *OM*_0_ and the *y*-axis be k, and the length from point *O* to the central axis be $d_{4}$. Based on this information, through the calculations between the coordinates of each point, the lengths of *HJ*_1_ and *HJ*_0_ can be determined by

(9) $$HJ_{1}=R_{0}\cos\left(\theta +w\right)-R_{2}\cos\left(\theta +h\right),$$

(10) $$HJ_{0}=d_{4}-R_{2}\cos\left(\theta +h\right).$$

In the triangle formed by points *H*_1_, *J*_1_, and *G*_1_, let the angle between the line *G*_1_*H*_1_ and the line *H*_1_*J*_1_ be δ, then

(11) $$\cos\delta =\frac{R_{0}\cos\left(\theta +w\right)-R_{2}\cos\left(\theta +h\right)}{R_{1}}.$$

In the triangle formed by points *H*_1_, *J*_0_, and *I*_0_, by simultaneously solving Equations (10) and (11), the value of $r_{\mathrm{mid}}$ is obtained as follows:

(12) $$r_{\mathrm{mid}}=\frac{H_{1}J_{0}}{\cos\delta }=\frac{R_{1}\left(d_{4}-R_{2}\cos\left(\theta +h\right)\right)}{R_{0}\cos\left(\theta +w\right)-R_{2}\cos\left(\theta +h\right)}.$$

In Figure 7b, the length of *M*_1_*K*_0_ is obtained as follows:

(13) $$M_{1}K_{0}=d_{4}-R_{3}\cos\left(\theta +k\right).$$

The length of *I*_0_*M*_1_ can be determined using the radius of Arc-II and the length of $r_{\mathrm{mid}}$,

(14) $$I_{0}M_{1}=\frac{R_{1}\left(d_{4}-R_{2}\cos\left(\theta +h\right)\right)}{R_{0}\cos\left(\theta +w\right)-R_{2}\cos\left(\theta +h\right)}+R_{4}.$$

The radius $r_{\min}$ can be calculated from the coordinates using Equation (13),

(15) $$r_{\min}=d_{4}-R_{3}\cos\left(\theta +k\right)-R_{4}.$$

In Figure 7a, draw auxiliary lines *OH*_2_ and *OI*_2_. *I*_2_*H*_2_ represents the maximum radius $r_{\max}$ of the object that the gripper can grasp. The coordinates of *I*_2_ can be determined as follows:

(16) $$I_{2}\left(R_{0}\sin\left(\theta +w\right),\;d_{4}\right).$$

The length of *G*_1_*I*_2_ can be calculated as follows:

(17) $$G_{1}I_{2}=R_{0}\cos\left(\theta +w\right)-d_{4}.$$

The value of $r_{\max}$ can be determined using the radius of Arc-I and Equation (17) as follows:

(18) $$r_{\max}=R_{1}-R_{0}\cos\left(\theta +w\right)+d_{4}.$$

The values of $r_{\min}$ and $r_{\max}$ obtained from Equations (15) and (18) represent the range of object radii that the gripper can grasp when the gripper rotation angle is θ.

### 3.3. Static Analysis of LCM Gripping Object

**Figure 8:**
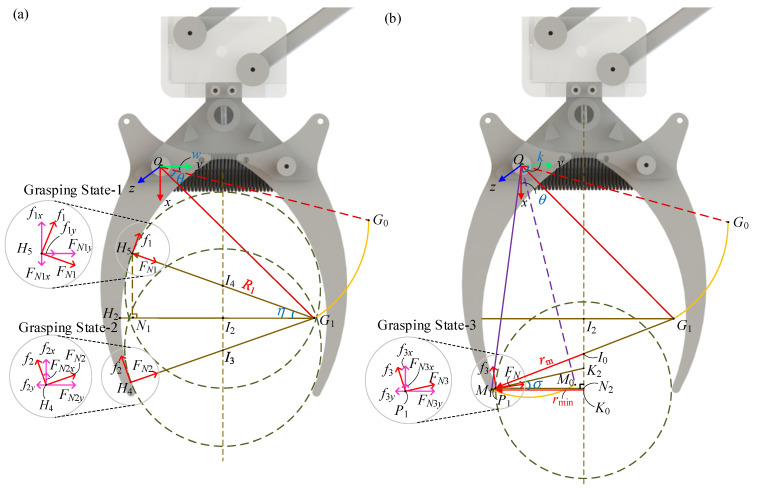
Diagram of the support and friction forces on the object in three gripping states by the gripper. (**a**) Force analysis diagrams of Grasping State-1 and Grasping State-2. (**b**) Force analysis diagrams of Grasping State-3.

In the LCM, there are two gripping modes: active and passive. Both exert the same direction of gripping force but differ in magnitude. This study investigates the relationship between the radius range and the mass of the object being gripped, while fixing the gripper’s rotation angle. A Cartesian coordinate system with the rotation center *O* as the origin is used to study the geometric characteristics of the gripper. The geometrical relationship between the points is shown in Figure 8.

During the process of the gripper grasping the object, the gripper itself experiences six forces: the pulling forces from the main tendon branches I and II ($T_{1}$ and $T_{2}$), the elastic force from spring-1 ($F_{d1}$), the gravity (G), the support force from the object ($F_{N}^{\prime }$), and the friction force from the object ($f^{\prime }$).

The friction and support forces exerted by the gripper on objects of different radii vary with the gripper angle during grasping. When the gripper grasps an object, there are three states:

In the first state, the center of the object is located above point *I*_2_, and the object is tangent to the gripper at Arc-I.

In the second state, the center of the object is located below point *I*_2_, and the object is tangent to the gripper at Arc-I.

In the third state, the center of the object is located below point *I*_2_, and the object is tangent to the gripper at Arc-II.

The support force and friction force acting on the object differ in these three states, thus the mass of the object that can be grasped also varies.

The support force and friction force acting on the object in each grasping state can be decomposed, as shown in Figure 8. Through the geometric relationships between the coordinates, we know the lengths of *G*_1_*I*_2_ and *I*_2_*I*_4_. Additionally, the length of *G*_1_*H*_5_ is $R_{1}$, and *I*_4_*H*_5_ represents the radius r of the object being grasped. Let the angle between the line *I*_2_*G*_1_ and the line *I*_4_*G*_1_ be η. Therefore, the sine and cosine values of η can be calculated as follows:

(19) $$\sin\eta =\frac{\sqrt{{\left(R_{1}-r\right)}^{2}-{\left(R_{0}\cos\left(\theta +w\right)-d_{4}\right)}^{2}}}{R_{1}-r},$$

(20) $$\cos\eta =\frac{R_{0}\cos\left(\theta +w\right)-d_{4}}{R_{1}-r},$$

The support force $F_{N1}$ and friction force $f_{1}$ in Grasping State-1 can be decomposed into their components along the *x* and *y*-axes, calculated using Equations (19) and (20). From the force analysis of the object in Grasping State-1, the force balance equations for the object can be established as follows:

(21) $$mg=2f_{1}\cdot \cos\eta -2F_{N1}\cdot \sin\eta .$$

Decompose the support force and friction force acting on the object in Grasping State-2, as shown in Figure 8a. Since *G*_1_*H*_4_ and *G*_1_*H*_5_ are symmetric about *G*_1_*H*_2_, the angle η is therefore equal to the angle formed by *I*_2_*G*_1_ and *I*_3_*G*_1_. Thus, the magnitudes of the support force $F_{N2}$ and the friction force $f_{2}$ decomposed along the *x* and *y*-axes are the same as in Grasping State-1, but their directions are different. Therefore, the force equilibrium equations for the object in Grasping State-2 are

(22) $$mg=2f_{2}\cdot \cos\eta +2F_{N2}\cdot \sin\eta .$$

When the object is in grasping state-3, the Arc-II of the gripper is tangent to the object. The force analysis for this state is shown in Figure 8b. In the triangle formed by points *K*_0_, *M*_1_ and *K*_2_, the length of *M*_1_*K*_2_ can be determined, and the length of *M*_1_*K*_0_ can be obtained. Therefore, the sine and cosine values of the angle σ formed by *K*_0_*M*_1_ and *M*_1_*K*_2_ can be derived:

(23) $$\sin\sigma =\frac{\sqrt{{\left(r+R_{4}\right)}^{2}-{\left(d_{4}-R_{3}\cos\left(\theta +k\right)\right)}^{2}}}{r+R_{4}},$$

(24) $$\cos\sigma =\frac{d_{4}-R_{3}\cos\left(\theta +k\right)}{r+R_{4}}.$$

The equilibrium equations for the forces acting on the object are

(25) $$mg=2f_{3}\cdot \cos\sigma +2F_{N3}\cdot \sin\sigma .$$

In Figure 8a, draw a perpendicular line from point *H_5_* to *G*_1_*H*_2_ with the foot of the perpendicular at *N*_1_. In the triangle formed by points *H*_5_, *G*_1_, and *N*_1_*,* the sine and cosine values of η can be obtained from Equations (19) and (20). The coordinates of point *G*_1_ are known, and the length of *G*_1_*H*_5_ is *R*_1_. Therefore, the lengths of *H*_5_*N*_1_ and *G*_1_*N*_1_ can be determined by

(26) $$H_{5}N_{1}=R_{1}\cdot \frac{\sqrt{{\left(R_{1}-r\right)}^{2}-{\left(R_{0}\cos\left(\theta +w\right)-d_{4}\right)}^{2}}}{R_{1}-r},$$

(27) $$G_{1}N_{1}=R_{1}\cdot \frac{R_{0}\cos\left(\theta +w\right)-d_{4}}{R_{1}-r}.$$

By solving Equations (26) and (27) simultaneously, the coordinates of *H*_4_ and *H*_5_ can be determined as follows:

(28) $$H_{4}\left(R_{0}\sin\left(\theta +g\right)+R_{1}\cdot \frac{\sqrt{{\left(R_{1}-r\right)}^{2}-{\left(R_{0}\cos\left(\theta +w\right)-d_{4}\right)}^{2}}}{R_{1}-r},\;R_{0}\cos\left(\theta +g\right)-R_{1}\cdot \frac{R_{0}\cos\left(\theta +g\right)-d_{4}}{R_{1}-r}\right),$$

(29) $$H_{5}\left(R_{0}\sin\left(\theta +g\right)-R_{1}\cdot \frac{\sqrt{{\left(R_{1}-r\right)}^{2}-{\left(R_{0}\cos\left(\theta +w\right)-d_{4}\right)}^{2}}}{R_{1}-r},\;R_{0}\cos\left(\theta +g\right)-R_{1}\cdot \frac{R_{0}\cos\left(\theta +g\right)-d_{4}}{R_{1}-r}\right),$$

When the object is in Grasping State-3, a perpendicular line is drawn from the tangent point between the object and the gripper to $K_{0}K_{2}$, with the foot of the perpendicular at *N*_2_. The sine and cosine values of σ are known from Equations (23) and (24), and the coordinates of point *K*_0_ are known. Therefore, by solving these equations simultaneously, the coordinates of point *P*_1_ can be determined by

(30) $$P_{1}\left(R_{3}\sin\left(\theta +k\right)-R_{4}\cdot \frac{\sqrt{{\left(r+R_{4}\right)}^{2}-{\left(d_{4}-R_{3}\cos\left(\theta +k\right)\right)}^{2}}}{r+R_{4}},\;R_{3}\cos\left(\theta +k\right)+R_{4}\cdot \frac{d_{4}-R_{3}\cos\left(\theta +k\right)}{r+R_{4}}\right).$$

Similarly, the coordinates of points *H*_5_ and *P*_1_ can also be derived using the above method of calculation. Let the angle between *N*_1_*S*_1_ and Plane-3 be γ. Draw a perpendicular line from point *S*_1_ to Plane-3, with the foot of the perpendicular at *S*_4_. In the triangle formed by points *N*_1_, *R*_1_ and *S*_4_, the sine and cosine values of γ can be determined. By means of the above information, he components of the tensile forces *T*_1_ and *T*_2_ of the main tendon branch rope along the *x* and *y* axes are

(31) $$T_{1x}=T_{2x}=T_{1}\cdot \cos\varepsilon =T_{1}\cdot \frac{\sqrt{{d_{6}}^{2}-{d_{5}}^{2}-{\left(d_{4}-r_{4}\cdot \cos\left(t-\theta \right)\right)}^{2}}}{d_{6}},$$

(32) $$T_{1y}=T_{2y}=T_{1}\cdot \sin\varepsilon \cdot \cos\gamma =T_{1}\cdot \frac{d_{4}-r_{4}\cdot \cos\left(t-\theta \right)}{d_{4}}.$$

The extension of spring-2, denoted as $\Delta x_{2}$, consists of two parts: the extension of the rope when the leg bends (Figure 1) and the length of *Q*_1_*Q* when the gripper grasps. Due to the relatively small length of *Q*_1_*Q* compared to the extension of the rope, it is negligible.

When the gripper is grasping the object and is in Grasping State-1, the torque balance equation with respect to point *O*, considering the forces acting on the gripper, can be expressed as

(33) $$\begin{array}{l} F_{d1}\cdot r_{2}\cdot \sin(b-\theta )+G\cdot r_{3}\cdot \cos(i-\theta )-\left(T_{1y}+T_{2y}\right)\cdot r_{4}\cdot \sin\left(t-\theta \right)+\\ \left(T_{1x}+T_{2x}\right)\cdot r_{4}\cdot \cos\left(t-\theta \right)-\left(f_{1x}^{\prime }-F_{N1x}^{\prime }\right)\cdot H_{5y}-\left(f_{1y}^{\prime }+F_{N1y}^{\prime }\right)\cdot H_{5x}=0 \end{array}.$$

By substituting (31) and (32) into (33), the value of $F_{N1}$ can be obtained. $F_{N1}$ and $F_{N1}^{\prime }$ are action and reaction forces, respectively; therefore,

(34) $$F_{N1}=F_{N1}^{\prime }=\frac{\left(\begin{array}{l} F_{d1}\cdot r_{2}\cdot \sin\left(b-\theta \right)+G\cdot r_{3}\cdot \cos\left(i-\theta \right)-\\ \left(T_{1y}+T_{2y}\right)\cdot r_{4}\cdot \sin\left(t-\theta \right)+\left(T_{1x}+T_{2x}\right)\cdot r_{4}\cdot \cos\left(t-\theta \right) \end{array}\right)}{\left(\mu \cos\eta -\sin\eta \right)\cdot H_{5y}+\left(\mu \sin\eta +\cos\eta \right)\cdot H_{5x}},$$

The value of the frictional force *f*_1_ exerted by the gripper on the object is

(35) $$f_{1}=f_{1}^{\prime }=\mu F_{N1}=\mu \cdot \frac{\left(\begin{array}{l} F_{d1}\cdot r_{2}\cdot \sin\left(b-\theta \right)+G\cdot r_{3}\cdot \cos\left(i-\theta \right)-\\ \left(T_{1y}+T_{2y}\right)\cdot r_{4}\cdot \sin\left(t-\theta \right)+\left(T_{1x}+T_{2x}\right)\cdot r_{4}\cdot \cos\left(t-\theta \right) \end{array}\right)}{\left(\mu \cos\eta -\sin\eta \right)\cdot H_{5y}+\left(\mu \sin\eta +\cos\eta \right)\cdot H_{5x}}.$$

By substituting Equations (34) and (35) into Equation(21), the mass $m_{1}$ of the object can be determined as

(36) $$m_{1}=2\cdot \frac{\left(\begin{array}{l} F_{d1}\cdot r_{2}\cdot \sin\left(b-\theta \right)+G\cdot r_{3}\cdot \cos\left(i-\theta \right)-\\ \left(T_{1y}+T_{2y}\right)\cdot r_{4}\cdot \sin\left(t-\theta \right)+\left(T_{1x}+T_{2x}\right)\cdot r_{4}\cdot \cos\left(t-\theta \right) \end{array}\right)}{g\cdot \left(\left(\mu \cos\eta -\sin\eta \right)\cdot H_{5y}+\left(\mu \sin\eta +\cos\eta \right)\cdot H_{5x}\right)}\cdot \left(\mu \cos\eta -\sin\eta \right).$$

Similarly, the grasping masses for Grasping State-2 and Grasping State-3 can be obtained as follows:

(37) $$m_{2}=2\cdot \frac{\left(\begin{array}{l} F_{d1}\cdot r_{2}\cdot \sin\left(b-\theta \right)+G\cdot r_{3}\cdot \cos\left(i-\theta \right)-\\ \left(T_{1y}+T_{2y}\right)\cdot r_{4}\cdot \sin\left(t-\theta \right)+\left(T_{1x}+T_{2x}\right)\cdot r_{4}\cdot \cos\left(t-\theta \right) \end{array}\right)}{g\cdot \left(\left(\mu \cos\eta +\sin\eta \right)\cdot H_{4y}-\left(\mu \sin\eta -\cos\eta \right)\cdot H_{4x}\right)}\cdot \left(\mu \cos\eta +\sin\eta \right).$$

(38) $$m_{3}=2\cdot \frac{\left(\begin{array}{l} F_{d1}\cdot r_{2}\cdot \sin\left(b-\theta \right)+G\cdot r_{3}\cdot \cos\left(i-\theta \right)-\\ \left(T_{1y}+T_{2y}\right)\cdot r_{4}\cdot \sin(m-\theta )+\left(T_{1x}+T_{2x}\right)\cdot r_{4}\cdot \cos\left(m-\theta \right) \end{array}\right)}{g\cdot \left(\left(\mu \cos\sigma +\sin\sigma \right)\cdot P_{1y}-\left(\mu \sin\sigma -\cos\sigma \right)\cdot P_{1x}\right)}\cdot \left(\mu \cos\sigma +\sin\sigma \right).$$

## 4. Experiment

### 4.1. Construction of the Drone and LCM Experimental Platform

**Figure 9:**
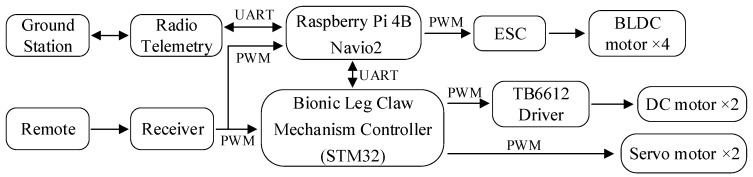
Hardware structure of the drone and LCM.

The drone’s control system is implemented using Emlid’s Navio2 and the Raspberry Pi 4B from the Raspberry Pi Foundation. The Navio2 is connected to the Raspberry Pi 4B, providing the essential sensors and interfaces required for flight control. The Raspberry Pi 4B functions as the onboard computer, running the Ardupilot flight control software and using the sensor data from Navio2 to execute flight control algorithms that manage the drone’s attitude, heading, and altitude. The control of the LCM is implemented using the STM32F103C8T6 microcontroller from STMicroelectronics, which drives the DC motor to operate the gripper via PWM signals and controls the servos to maintain balance during perching. Communication between the flight control system and the STM32F103C8T6 is established via UART. The hardware structure of the drone and LCM is shown in Figure 9.

Additionally, we selected the DJI F450 frame for the drone, with a net weight of 282 g. The electronic speed controllers (ESCs) used are Hobbywing 20A brushless ESCs, and the brushless DC motors chosen are Yuanhang X2216 KV950. When paired with the APC1047 propellers from APC Propellers, each motor can achieve a maximum thrust of 9 N. The drone is powered by a 3S lithium battery from Quansheng Electronics. We use the N20 DC gear motor from Kejin for driving the lead screw above the hip, which opens the bottom gripper. The gear ratio of this motor is 1:603, and the time required for the gripper to transition from the closed state to the open state is 100 ms. The hip joint uses the MG995 metal servos from TowerPro, which drive the leg rotation during grasping tasks. The servo weighs 45 g and has a maximum torque of 13 kg·cm. The stiffness of the spring at the gripper (spring-1) is 180 N/m, and the stiffness of the spring at the hip (spring-2) is 340 N/m. The total weight of the drone is 1332 g, with the leg-claw mechanism weighing 402 g. After integrating the leg-claw mechanism with the drone, the LCM accounts for 23% of the total mass. This weight distribution demonstrates a well-balanced design where the leg-claw mechanism is lightweight relative to the overall drone, ensuring that the addition of the LCM does not significantly compromise the drone’s flight performance.

### 4.2. The Experiment of Opening and Closing the Bistable Gripper

**Figure 10:**
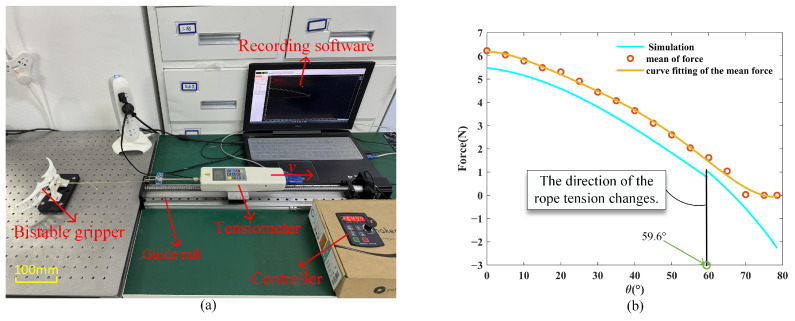
Experimental of the gripper opening angle and required force. (**a**) Experimental equipment. (**b**) Experimental results.

To validate the established static model of the force required to transition the bistable gripper from a closed state to an open state, we designed an experiment to analyze the forces involved in opening the gripper. The experimental setup is shown in Figure 10a. The main experimental equipment includes a standalone bistable gripper, a guide rail, a tensiometer, data recording software, and a guide rail controller.

The gripper is connected to a tensiometer via a rope, and during the pulling process, the force software records the tensiometer data in real time. Since it is difficult to measure the gripper’s angle during opening, the opening angle is converted into the tensiometer’s displacement. The displacement of the tensiometer corresponds to the change in the length of the rope’s branch, and the change in the gripper angle can be inversely solved through Equation (5) At the start of the experiment, ensure that the rope is straight and in a critical taut state, and record the initial position displayed on the guide rail controller. When the tensiometer’s reading returns to zero, pause the movement of the guide rail and record the final position of the tensiometer displayed on the guide rail controller. At the beginning of the experiment, there is an initial pre-tension in the rope, causing the tensiometer reading to increase without changing the gripper’s angle. Once the rope’s tension reaches the minimum force required to open the gripper, the gripper’s angle starts to change. As the gripper’s opening angle increases, the tensiometer reading decreases until it reaches zero.

We conducted ten experiments to open the gripper and combined the data from each experiment to generate the force-angle characteristic curve. The experimental results are shown in Figure 10b. The gripper opening experiment was conducted 10 times. The blue curve represents the simulation results of the mathematical model, the orange dots represent the average force values during the experiments, and the yellow curve is the fitted curve of the average values.

As illustrated in Figure 10b, a deviation exists between the actual and simulated values. This discrepancy primarily stems from the frictional forces encountered during the gripper’s opening process. In the experiment, the rope branch facilitating the gripper’s opening passes through a cable hole and connects to a tensiometer. As the tensiometer moves to the right, friction develops between the rope and the cable hole due to the tension in the rope. Additionally, friction occurs between the gripper and the connecting plate during the opening process. As the opening angle of the gripper increases and the required force diminishes, the frictional forces generated during this process gradually decrease. Initially, the force exerted by spring-1 acts as resistance to the gripper’s opening. Once this force exceeds the rotation center of the gripper, it transitions into the driving force for the opening mechanism. At this point, the tensiometer reading steadily declines until it reaches zero. The discontinuity observed in the simulation values arises because, at this angle, the torque direction of the opening claw’s rope branch changes, resulting in a sudden alteration in the rope’s force.

**Figure 11:**
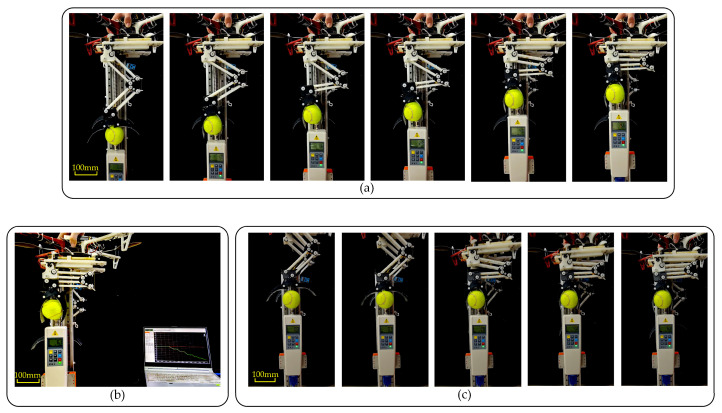
Bistable gripper triggering experiment: (**a**) Triggering force when a ball impacts the claw at a constant speed to initiate grasping. (**b**) Experimental equipment. (**c**) Triggering force when the drone accelerates and impacts the ball during a controlled descent.

We also measured the impact force required for the gripper to close from an open state during passive grasping. In the experiment, a pressure sensor was fixed on a sliding block, with a small ball attached to the front of the sensor. The experiment included two scenarios: first, the sliding block was controlled to move at a constant speed to collide with the small ball; second, a drone was used to perform a natural descent motion to collide with the small ball. These scenarios simulated the process of an object colliding with the LCM claw under different motion modes. By recording the pressure exerted when the ball contacted the claw, the impact force required for the gripper to close was precisely measured. To ensure data reliability, multiple repeated experiments were conducted, and the impact force required for each grasp was recorded. The experimental setup and results are shown in Figure 11.

The experimental results indicate that the impact force required to trigger the grasping action is nearly identical for both constant-speed motion and accelerated collisions. All experimental data show that the triggering force for grasping is approximately 0.8 N. This value is relatively small for a passive grasping mechanism, and the impact force increases to approximately 5.8 N as the leg fully folds. Compared to existing passive grasping mechanisms, the passive gripper design in [28] requires tens of newtons of impact force to trigger the grasping action. In contrast, the LCM’s lower triggering force not only reduces the initial velocity requirement for external objects but also broadens its application scenarios. For instance, it enables effective grasping of lightweight or flexible objects without applying excessive force, thereby improving both the success rate and adaptability of the grasping process.

The design of the LCM exhibits significant advantages in terms of low energy consumption and low triggering force during the opening and closing process. First, the maximum force required to open the gripper is approximately 6 N, which is much lower than that of traditional designs. This reduced opening force effectively reduces the motor power requirements, significantly lowering energy consumption and improving the drone’s endurance. Secondly, the lower closing force exerts less pressure on the target object, making the LCM gripper more suitable for tasks such as grasping and perching, especially when handling delicate or flexible objects, as it avoids applying excessive force that could damage the object.

### 4.3. LCM Load-Bearing Capacity Test

In the previous section, we calculated the maximum weight the LCM can grasp. In this section, static grasping tests and active grasping tests are conducted to validate the theoretical findings. Due to the difficulty in measuring the gripper’s opening angle, we marked two points on the gripper and converted the distance between them into the angle change during the gripper’s opening. The conversion relationship can be given as

(39) $$\theta =\arcsin\frac{L-x}{2r},$$

where *L* is the distance between the two marked points, *r* is the distance from the marked points to the rotation center of the gripper, and *x* is the distance between the two marked points when the gripper is in the closed state.

**Figure 12:**
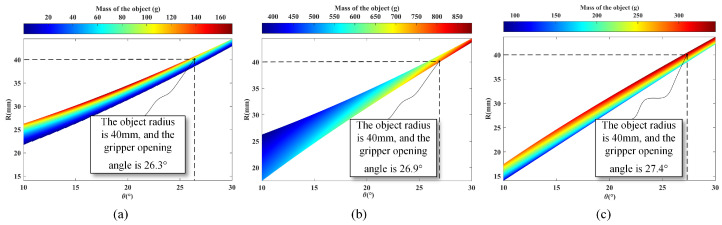
Relationship diagram of gripper opening and closing angles, object radius, and maximum grasped object mass in three Gripping States. (**a**) Grasping State-1; (**b**) Grasping State-2; (**c**) Grasping State-3.

**Figure 13:**
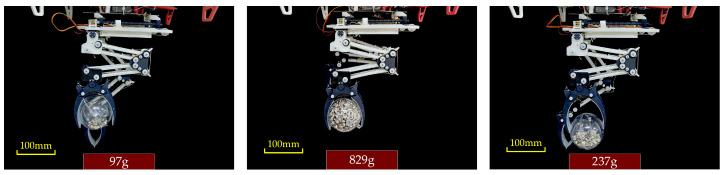
Experimental diagram of grasping mass in three grasping states.

In the static grasping experiment, we assessed the LCM’s ability to grasp objects of different weights in various grasping states. As shown in Figure 12 and Figure 13, the LCM was configured into three distinct grasping states, and tests were conducted for each configuration. During the experiment, we accurately measured the positions of the marked points on the gripper and used Equation (39) to calculate the opening angle for each state. Additionally, we measured the actual weight of the objects grasped by the gripper and compared these values with the theoretical calculations.

For instance, in one grasping state, the gripper was able to hold a ball with a radius of 40 mm, achieving an actual grasping weight of 97 g, while the theoretical calculation was approximately 100 g. Using the same method, we measured the weight of the ball in the second and third grasping states, with actual grasped weights of 829 g and 237 g, respectively, compared to theoretical values of around 850 g and 250 g. The results indicate that the actual grasped weights were very close to the simulated values. The slight discrepancies may have been caused by the gripper legs not fully bending under the weight of the object, preventing the main tendon from stretching as anticipated. To avoid overloading the mechanism, we ensured that the weight of each object remained within the system’s safe load capacity, thus keeping the experimental values as close as possible to the theoretical predictions.

**Figure 14:**
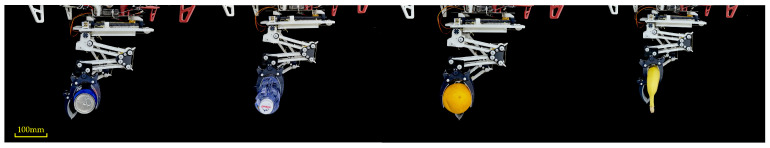
Schematic diagram of grasping everyday objects.

Additionally, we assessed the LCM’s ability to grasp a variety of everyday objects. The results demonstrated excellent performance, stability, and gripping force, with the mechanism successfully securing objects of different shapes and sizes, such as bottles and fruits, while maintaining their stability, as shown in Figure 14.

Undoubtedly, the silicone layer on the claws plays a critical role in ensuring the success rate of grasping. The 3D-printed materials used in the LCM structure have a relatively low friction coefficient. The friction performance of bird claws is influenced by multiple factors, such as the surface roughness of the claw and the object it contacts, the degree of curvature of the claw, and the pressure exerted by the claw. According to experimental data from references [33,34], the static friction coefficient between keratinized bird claws and rough surfaces, such as tree bark, typically ranges from 0.3 to 0.6. This range is inferred from studies on the friction of rough surfaces similar to tree bark. Raptors (e.g., eagles and falcons) tend to have claws with higher friction properties, as their claw surfaces are rougher and sharper, with friction coefficients potentially reaching 0.5 to 0.8 when interacting with tree bark or wooden surfaces. Silicone is known for its high surface friction coefficient, and compared to rigid claw structures, it enhances the static friction between the claw and the object’s surface. This increased friction effectively prevents the object from slipping during the grasping process due to external forces.

In our theoretical model, the friction coefficient is a key variable. In this study, we employed a fixed friction coefficient value of μ = 0.6. In future research, we plan to thoroughly investigate the physical properties of bird claw soft tissues, including their structural and mechanical characteristics. We will also seek to optimize the design of silicone materials from a biomimetic perspective and explore the impact of varying friction coefficients on grasping stability using dynamic theoretical models.

Overall, the LCM demonstrated excellent grasping stability and gripping force when handling objects of different shapes and sizes. The results of the static grasping experiment indicate that the LCM claw not only performed well in theoretical calculations but also successfully completed tasks stably in practical operations. Although there were some minor discrepancies, these deviations were within the system’s safe load capacity and did not affect its overall performance. This provides reliable experimental support for the LCM in a wide range of application scenarios.

### 4.4. LCM Active Gripping Experiment

**Figure 15:**
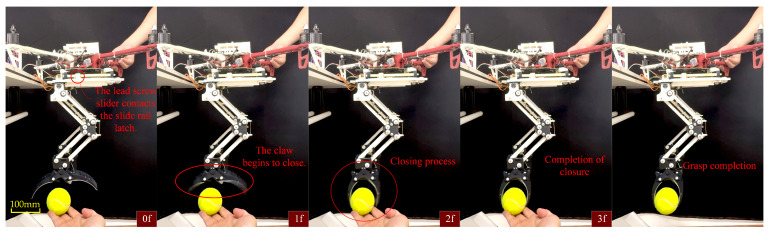
Frame-by-frame analysis of the LCM’s active grasping process.

In the active grasping experiment, we achieved the gripper’s closure by controlling the movement of the screw slider. Specifically, control system commands were used to move the screw slider forward along the lead screw. When the slider reached a specific position, it triggered the unlocking of the sliding track latch, releasing the locking mechanism of the gripper. At this point, the gripper closed reliably under the force of the hip spring, tightly grasping the target object, as illustrated in Figure 15.

To more clearly demonstrate the functional and performance advantages of active grasping, we designed an experiment to compare the success rate of the LCM when grasping soft objects. The experiment focused on both active and passive grasping, with test objects including soft items such as feathers and tomatoes.

**Figure 16:**
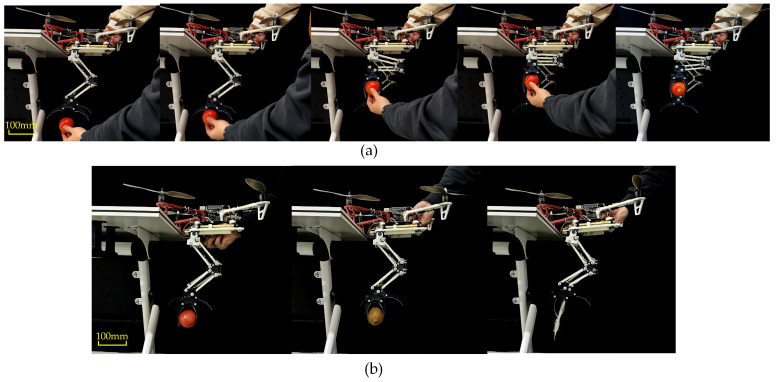
(**a**) The passive grasping process of the tomato. (**b**) Active grasping process.

In the passive grasping experiment, the drone and the leg-claw mechanism were fixed in place, and objects were given an initial velocity to collide with the LCM, triggering the claws to close and grasp the object. For active grasping, the experimental method was as previously described, with the grasping action completed through active control of the mechanism. During the experiment, we recorded the success rate of each grasping attempt. The criteria for successful grasping included the degree of deformation of the object and the stability of the grasp. For each grasping method and each type of object, 20 trials were conducted. The experimental process is shown in Figure 16, and the results are shown in Table 1.

The results showed that for tomato grasping, 5 trials failed. The main reason for failure was the soft surface of the tomato, which made it more prone to surface damage during passive grasping, leading to grasping failure. The main cause of failure in kiwi grasping was similar. For feathers, which are lightweight and soft, even when given high velocity and acceleration, passive grasping often failed to trigger an effective grasping action. This is a common issue with many similar passive grasping mechanisms. In contrast, active grasping achieved effective grasping through precise control. Although the gripping force of active grasping was slightly smaller than that of passive grasping, the success rate of active grasping was significantly higher when handling soft objects such as feathers and tomatoes.

This advantage also extends to other fragile objects, such as eggs and glass bottles. In passive grasping, these objects often break due to excessive force or inadequate control, resulting in grasping failure. Active grasping, however, effectively addresses these issues by enabling precise adjustments to the grasping action.

In conclusion, the experimental results confirm that active grasping mechanisms provide significantly higher success rates and stability compared to passive grasping when handling soft and fragile objects, demonstrating notable performance advantages. Therefore, compared with those mechanisms with only passive grasping mechanisms [22,23,24,27,28], our active grasping mechanism is a major advantage in handling these soft objects. Similarly, compared with those mechanisms with active grasping mechanisms [11,12], the active grasping mechanism of LCM is faster.

In addition to the advantages demonstrated by active grasping mechanisms, the performance of the LCM is further enhanced by the contribution of key auxiliary components, particularly the springs integrated within the mechanism. These springs play a critical role in improving both the efficiency and reliability of the LCM during grasping tasks. Spring-1, located in the claw, enables the claw to close rapidly during grasping while providing the primary gripping force. Since the claw must overcome the tension of Spring 1 when opening, a spring with an elasticity coefficient of 180 N/m was selected. This elasticity coefficient ensures that the claw can open smoothly while providing sufficient gripping force, balancing operational flexibility and gripping capability. Spring-2, positioned on the top plate, further enhances the gripping force of the claw. Directly connected to the lead screw slider, Spring 2 achieves better force transmission. Therefore, a spring with a higher elasticity coefficient of 340 N/m was selected. This design provides greater stability during both grasping and perching, ensuring reliable performance under various operating conditions. Additionally, the claw structure was designed with consideration for objects of varying sizes and shapes, enabling enveloping grasps of objects with a radius of approximately 40 mm. It also has the capability to lift objects weighing 4.12 times the weight of a single leg.

Through the coordinated action of Springs 1 and 2, along with a well-designed claw structure, the gripping performance of the LCM has been significantly improved. This improvement was validated through experiments, where the LCM achieved a grasping success rate of over 95% and effortlessly handled objects weighing 4.12 times the weight of a single leg.

### 4.5. Perching Experiment

**Figure 17:**
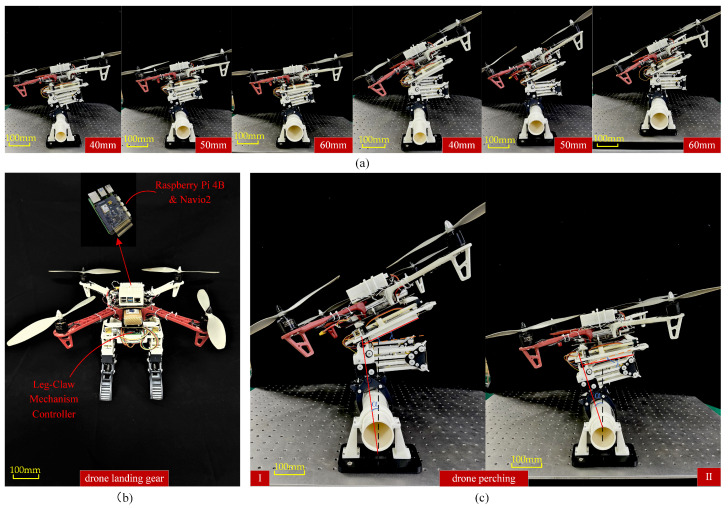
Perching experiment. (**a**) Drones perching on rods of different diameters. (**b**) Leg-claw mechanism as landing gear. (**c**) Comparison of drone perching leg angles.

When integrated with the drone, the LCM can function as the landing gear. The state of the mechanism when used as landing gear is shown in Figure 17b. To verify the perching capability of the LCM, the drone with the integrated LCM was tested for stability while perching on poles of different diameters (40 mm, 50 mm, and 60 mm), as shown in Figure 17a. When the claw part of the LCM contacts the perching surface, the claws quickly close passively. The legs are bent and folded by the weight of the drone. The unlocking rope connected to the main tendon unlocks the slide rail latch when the main tendon is stretched, allowing hip spring-2 to quickly contract and tighten the main tendon. The grip force of the claws is further increased. Sometimes the grip force of the claws alone is insufficient to achieve stable perching. In such cases, the drone’s attitude needs to be adjusted using the servos in the hip section. The servos’ rotation causes the LCM’s hip plate to rotate with the drone, as shown in Figure 17c. In this figure,α represents the angle between the leg and the centerline of the perching object, while λ represents the angle between the hip and the leg. For poles of different diameters, the LCM adjusts the angle λ between the leg and the hip section accordingly. Additionally, λ is influenced by the angle α between the leg and the centerline of the perching surface. When α increases, the center of gravity of the drone and the LCM moves closer to the centerline of the perching object, resulting in a smaller λ. Conversely, when α decreases, the servo motor adjusts the angle λ to ensure the center of gravity of the drone and the LCM remains close to the centerline of the perching object.

This experiment successfully validated the superior performance of the LCM in the perching and landing processes. It demonstrated the ability to achieve stable perching on different rod diameters and, through the coordinated operation of the servos and spring system, provided sufficient gripping force and attitude adjustment capabilities to ensure the drone’s stability. This provides reliable technical support for the drone’s multifunctional applications (perching, landing).

## 5. Conclusions

We designed and validated a biomimetic leg-claw mechanism (LCM) for drones, inspired by the bionic principles of birds, aiming to enhance the endurance and aerial interaction capabilities of rotor drones.

Firstly, the claw part of the LCM features a bistable gripper, which can passively close through external impact forces or actively close by unlocking the sliding clasp with a screw slider, enabling flexible grasping and perching actions. Additionally, inspired by the Automatic Digital Flexor Mechanism (ADFM) of birds, we integrated a ratchet-pawl device into the LCM’s knee joint. This device locks the leg in place when bent, ensuring stable gripping force. Secondly, we developed a static model to predict the force required to open the gripper and experimentally tested the impact force required for passive grasping. We also established a static model describing the relationship between the gripper angle, object radius, and grasping position to verify the maximum load capacity of the LCM. Furthermore, we analyzed the application scenarios for both active and passive grasping, providing customized grasping strategies for different objects. Finally, the LCM was installed on a drone and tested for perching adaptability on objects of varying sizes. Experimental validations of the models and designs showed that this biomimetic LCM not only has high load capacity but also enables stable and reliable grasping and perching on different targets, significantly enhancing the drone’s versatility in multi-scenario applications.

Future research will focus on the implementation of robotic vision to facilitate the drone’s autonomous perching and grasping of typical targets. In addition, the study in [35] provides insights for our gripper, particularly in enhancing response speed and energy conversion efficiency. The design in [36] also offers ideas for improving the adaptability of our biomimetic leg-claw mechanism in different operational modes, allowing us to envision its application in various environments.

## Figures and Tables

**Table 1:** Success rate test experiment for passive and active grasping.

Experimental Materials	Passive Grasping Success Rate	Active Grasping Success Rate
Tomato	75%	100%
Kiwi	80%	100%
Feather	-	100%

## Data Availability

The raw data supporting the conclusions of this article will be made available by the authors upon request.
